# Effects of terlipressin infusion during hepatobiliary surgery on systemic and splanchnic haemodynamics, renal function and blood loss: a double-blind, randomized clinical trial

**DOI:** 10.1186/s12871-019-0779-6

**Published:** 2019-06-15

**Authors:** Magdy Mohammed Mahdy, Mostafa Samy Abbas, Emad Zarief Kamel, Mohamed Fathy Mostafa, Ragaa Herdan, Shimaa Abbas Hassan, Ramy Hassan, Ahmed M. Taha, Tameem M. Ibraheem, Bashir A. Fadel, Mohammed Geddawy, Jehan Ahmed Sayed, Osama Ali Ibraheim

**Affiliations:** 1 Anesthesia and intensive care medicine, Faculty of Medicine, Assiut University, Assiut, 71515 Egypt; 2 Hepatobiliary surgery, Faculty of Medicine, Assiut University, Assiut, 71515 Egypt; 3 King Faisal Specialist Hospital & Research Center, Riyadh, Saudi Arabia

**Keywords:** Terlipressin, Portal pressure, Hemodynamics, Blood loss

## Abstract

**Background:**

Terlipressin, in general, is a vasopressor which acts via V1 receptors. Its infusion elevates mean blood pressure and can reduce bleeding which has a splanchnic origin. The primary outcome was to assess the impact of intraoperative terlipressin infusion on portal venous pressure during hepatobiliary surgery; the 2ry outcomes included effects upon systemic hemodynamics, estimated blood loss, and postoperative renal functions.

**Methods:**

This prospective randomized study involved 50 patients undergoing hepatobiliary surgery who were randomly and equally allocated into terlipressin group, or a control group. The terlipressin group received an initial bolus dose of (1 mg over 30 min) followed by a continuous infusion of 2 μg/kg/h throughout the procedure and gradually weaned over the first four postoperative hours, whereas the control group received the same volumes of normal saline. The portal venous pressure changes were measured directly through a portal vein angiocatheter.

**Results:**

Portal pressure was significantly reduced over time in the terlipressin group only (from 17.88 ± 7.32 to 15.96 ± 6.55 mmHg, *p* < .001). Mean arterial blood pressure was significantly higher in the terlipressin group. Estimated blood loss was significantly higher in the control group than the terlipressin group (1065.7 ± 202 versus 842 ± 145.5 ml; *p* = 0.004), and the units of packed RBCs transfused were significantly higher in the control group ((0–2) versus (0–4) *p* = 0.003). There was no significant difference between groups as regards the incidence of acute kidney injury.

**Conclusion:**

Intraoperative infusion of terlipressin during hepatobiliary surgery was shown to improve intraoperative portal hemodynamics with subsequent reduction in blood loss.

**Trial registration:**

Clinical trial number and registry URL: Trial registration number: NCT02718599. Name of registry: ClinicalTrials.gov. URL of registry: https://clinicaltrials.gov/ct2/show/NCT02718599. Date of registration: March 2016. Date of enrolment of the first participant to the trial: April 2016.

## Background

Intraoperative blood volume loss as little as 10 to 15% could result in splanchnic hypoperfusion, which usually persists during the period of hypovolemia [1]. This results in an intramucosal acidosis of the gut leading to a cascade of events that impair postoperative gastrointestinal function and cause complications [2–4]. Avoidance of hypotension during anesthesia, as much as possible, during such operations by adequate fluid replacement and or vasopressor support can positively affect the outcome [5, 6]. Recent studies suggest that cardiac output guided hemodynamic therapy may have particularly favorable effects on splanchnic perfusion and renal function and protects against postoperative organ dysfunction [1–8]. Some studies denoted that the use of vasopressors in hemodynamic optimization mostly offered beneficial effects [9].

Terlipressin is a synthetic analog of arginine vasopressin but with longer duration of action. It has been utilized in the management of circulatory dysfunction after paracentesis, hepatorenal syndrome and esophageal variceal bleeding in end-stage liver disease patients [10]. Terlipressin works through vasopressin-1 (V1) receptors which induce vasoconstriction with a subsequent decrease in the portal pressure and enhancement of renal blood flow [11]. We hypothesize that the terlipressin induced splanchnic vasoconstriction could reduce portal pressure, decrease blood loss, and improve postoperative renal function.

The primary aim of our study was to evaluate the impact of intraoperative terlipressin infusion on the hepatic hemodynamics (portal venous pressure). Secondary goals included the effects of terlipressin infusion upon intraoperative systemic hemodynamics, estimated blood loss during open hepatobiliary surgery, and the early postoperative renal function.

## Methods

This prospective, randomized, controlled study was approved by the Medical Ethics Committee at Assiut University (reference number IRB17200284). All participants provided written informed consent. The trial was registered prior to patient enrollment at ClinicalTrials.gov (NCT02718599). The study was carried out at Assiut University Hospitals, Al Rajhy Liver Hospital, between April 2016 and July 2017 in accordance with the Consolidating Standards of Reporting Trials (CONSORT) 2010 statement.

Eligible participants were adult patients aged > 18 years old with American Society of Anesthesiologists (ASA) Classification (Class I-II) undergoing major elective hepatobiliary surgery. Exclusion criteria included patients with preoperative renal failure, severe liver dysfunction (Child-Turcotte-Pugh grade C), hyponatremia (Na^+^ < 132 mmol/l), severe valvular heart disease, heart failure, symptomatic coronary heart disease, bradycardic arrhythmia (heart rate < 60/min), peripheral artery occlusive disease (clinical stadium II-IV), uncontrolled arterial hypertension (Blood pressure > 160/100 mmHg despite intensive treatment), pregnancy and intraoperative need for Pringle maneuver. Fifty patients were randomly and equally allocated into one of two groups, control group (C; *n* = 25) and terlipressin groups (T; n = 25) by simple randomization technique (sealed opaque envelopes) done by a study coordinator, who also encodes the drugs with matching random numbers. Surgeon, his assistants, and outcome assessing physician were kept blind to the grouping. All cases recruited to the study were done by the same surgeon.

All patients received standardized premedication with midazolam (0.02 mg/kg) and atropine (0.5 mg I.V). Anesthesia was induced in all patients with propofol (2 mg/kg) and fentanyl (1 μg/kg). Muscle relaxation for intubation was achieved by rocuronium (0.6–0.8 mg/kg) in both groups. Anesthesia was maintained with sevoflurane 1 MAC in medical air oxygen mixture (fraction of inspired oxygen 0.5), fentanyl infusion (1 μg/kg/hr.) and rocuronium infusion (0.01–0.012 mg/kg/min). Mechanical ventilation was controlled through a tidal volume of 6 to 8 mL/kg, and the ventilator rate (8–12/min) was adjusted to maintain an end-tidal CO2 of 35 to 40 mmHg. We used no PEEP with recruitment maneuvers repeated every 30 min after tracheal intubation. Each recruitment maneuver consisted of applying a continuous positive airway pressure of 30 cm of water for 30 s. Intraoperative normothermia was attained using warm intravenous fluids, a warm blanket, and humidifier. A 3-lm central venous catheter was placed through the right internal jugular vein, and central venous pressure (CVP) was monitored to guide fluid management during parenchymal dissection of the liver through maintaining CVP below 10 mmHg. A 22-gauge angiocatheter was inserted into the radial artery (after performing modified Allen’s test) and connected to the FloTrac/Vigileo™ monitor (software version 1.14; Edwards Life sciences, Irvine, CA, USA) for monitoring of cardiac output (COP), cardiac index (CI), stroke volume variation (SVV), and systemic vascular resistance (SVR). All blood pressure measurements were referenced to the level of the mid-axillary line. After surgical exposure of the portal vein, a 22-gauge angiocatheter was inserted into the portal vein and connected to a pressure transducer to measure the portal pressure at scheduled times, and for blood gas analysis of portal venous blood.

In a separate room, syringes containing Terlipressin for group T, and NaCl as a placebo for group C were prepared by an anesthesia technician who was not involved in the study after opening the sealed envelope and identification to which group the patient was to be enrolled. In group T, terlipressin was started just after exposure of the portal vein and obtaining a basal portal pressure reading, as an initial loading dose of 1 mg over 30 min followed by a continuous infusion of 2 μg/kg/hr. throughout the procedure and gradually weaned over the first 4 h postoperatively. In group C, patients received the same volume of normal saline. Intraoperative basal fluid replacement was attained in both groups through infusion of crystalloids (8 ml/kg/hr.), additional boluses of colloid solution in a dose of 3 ml/kg (Human Albumin 5% solution) were given when SVV was raised above 10% (a sustained rise during the previous 5 minutes) or in case of positive response (cardiac index increases above 10%) to previous fluid challenge. An infusion of dobutamine was initiated to raise the low cardiac output state conditions (cardiac index less than 2.5 l/min/m^2^) after appropriate fluid administration, and the goal was to maintain CI between 2.5 and 4 l/min/m^2^. Ephedrine boluses (5–15 mg), or norepinephrine infusion was allowed in addition to colloid blouses to correct a fall in systolic arterial pressure < 90 mmHg, or mean arterial pressure < 65 mmHg to maintain it above 70 mmHg and the systemic vascular resistance above 600 dyn. s/cm^5^. Transfusion of packed RBCs was allowed if the hemoglobin level has diminished below 7 g/dl. All patients were transferred postoperatively to the post-anesthesia care unit.

Portal blood pressure was measured after surgical exposure of the portal vein then every 30 min throughout the operation. Portal venous blood gas was reported regarding pH, partial pressure of CO2, and lactate level initially as baseline values, then intraoperatively starting with the terlipressin infusion or the placebo, then every 1 hour until the end of anesthesia, and collectively will be expressed as a mean value. Another intraoperative data collection was done 15 min after anesthesia induction, then every 30 min till the end of operation included heart rate (HR), mean arterial blood pressure (MAP), CVP, CI, SVV, SVR, and urine output (UOP). Fluid input (crystalloids, colloids, packed red blood cells, fresh-frozen plasma), and output (UOP, blood loss by calculating surgical sponges, suction canisters and the cell salvage device (if used)) were recorded. The mean postoperative hemodynamic data (HR, MAP, and UOP) were recorded from the averaged 24 h’ measurements of the first day after surgery. Conventional renal function, hemoglobin level (Hb), sodium, and potassium levels were also reported preoperative as a baseline and daily for the first three postoperative days. The incidence of renal impairment was calculated according to AKIN criteria.

### Sample size

Power analysis was performed on the level of portal pressure (the 1ry outcome). A previous study showed that infusion of terlipressin was accompanied by a significant reduction of portal pressure from 25.3 ± 3.2 mmHg to 21.1 ± 3.3 mmHg (*p* = .0001). Based on that literature [12], we considered that 20% reduction of portal pressure to be clinically relevant, assuming population size = 1000, confidence level = 95%, beta error = 0.2 and confidence interval = 0.109. Thus, 50 patients undergoing elective hepatobiliary surgery were included.

### Statistical analysis

Data were presented as a mean ± standard deviation for parametric data, median for non-parametric data, ratios, and percentages as appropriate. Continuous data with normal distribution were compared by paired or unpaired t-tests, whereas non-normally distributed data were assessed using the Mann-Whitney U test and Wilcoxon rank-sum test for unpaired and paired results, respectively. Chi-square test measured the association between qualitative variables. Statistical analysis was established using SPSS® version 16.0 (SPSS Inc., Chicago, IL, USA) for Windows®. A *P*-value of < 0.05 was considered to be statistically significant.

## Results

**Fig. 1:**
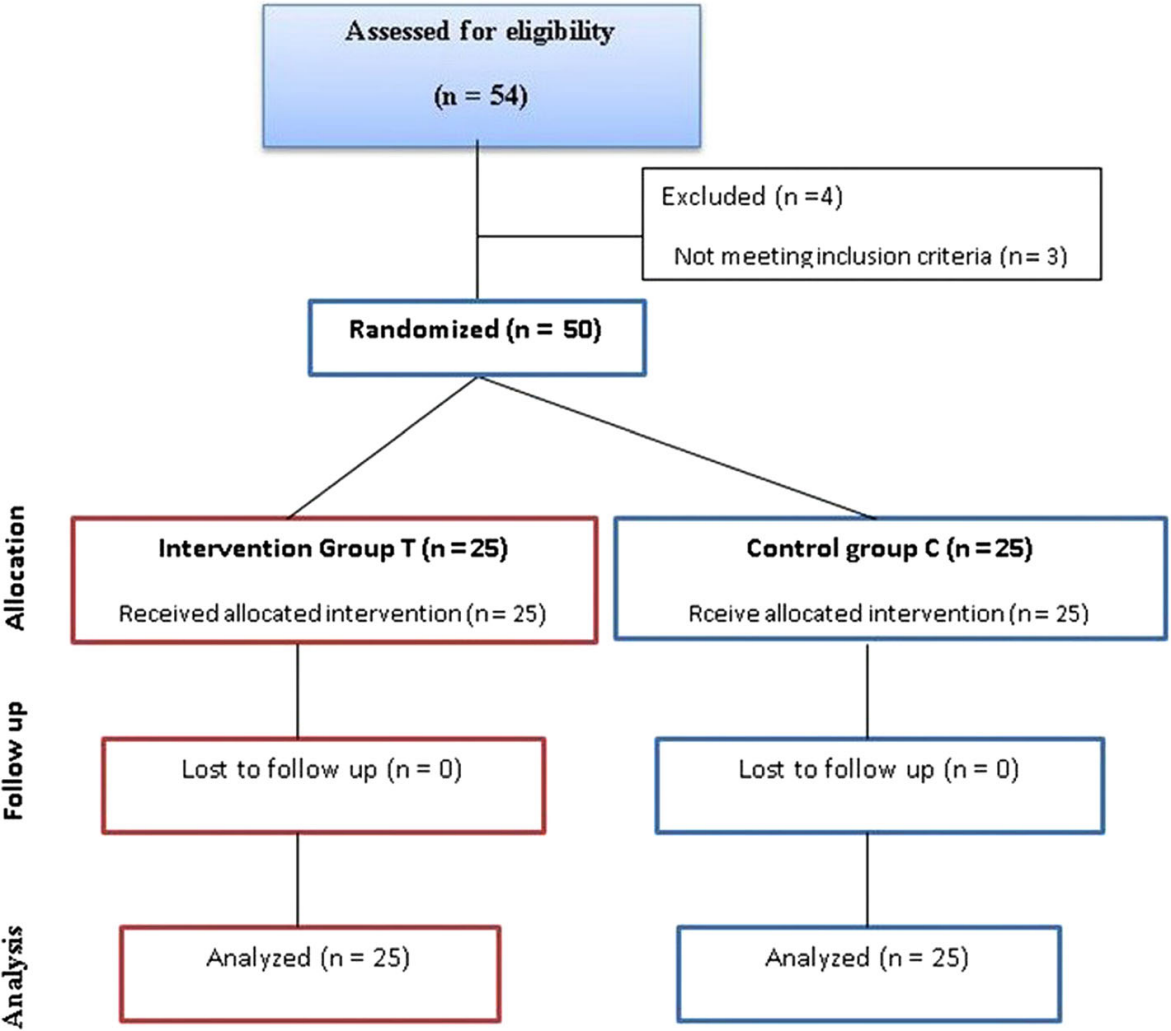
CONSORT flow diagram

A total of 50 patients were enrolled in this study and randomly allocated to the two groups as shown in the CONSORT flow-chart (Fig. 1). There were no significant differences between both groups regarding demographic data, clinical and perioperative characteristics, co-morbid risk factors, surgery type or its duration, nor the Intensive Care Unit (ICU) stay days (Table 1).

**Table 1:** demographic, clinical, and surgical variables

Variables	Group T N = 25	Group C N = 25	*P* value
Age (years)	58.7 ± 5.9	55.5 ± 8.4	0.2
Gender (male/female)	10/15	12/13	0.7
BMI (Kg/m^2^)	25.4 ± 3.6	24.8 ± 4.2	0.6
ASA N (%)			
ASA I	2 (8%)	3 (12%)	0.6
ASA II	23 (92%)	22 (88%)	
Surgery type			
Liver resection	13 (52%)	14 (56%)	0.8
Right hepatectomy	6	5	
Left hepatectomy	7	9	
Whipple’s operation	12 (48%)	11 (44%)	
Operative duration (hours)	6 (4–8)	6 (3–9)	0.51
ICU stay (days)	1 (1–3)	2 (1–4)	0.23
In hospital mortality (N)	0/25	1/25	0.31

Data are presented as mean ± SD, median (range) or number (%). BMI Body mass index, ASA American society of anesthesiologists. *P*-value < 0.05 was considered statistically significant

**Fig. 2:**
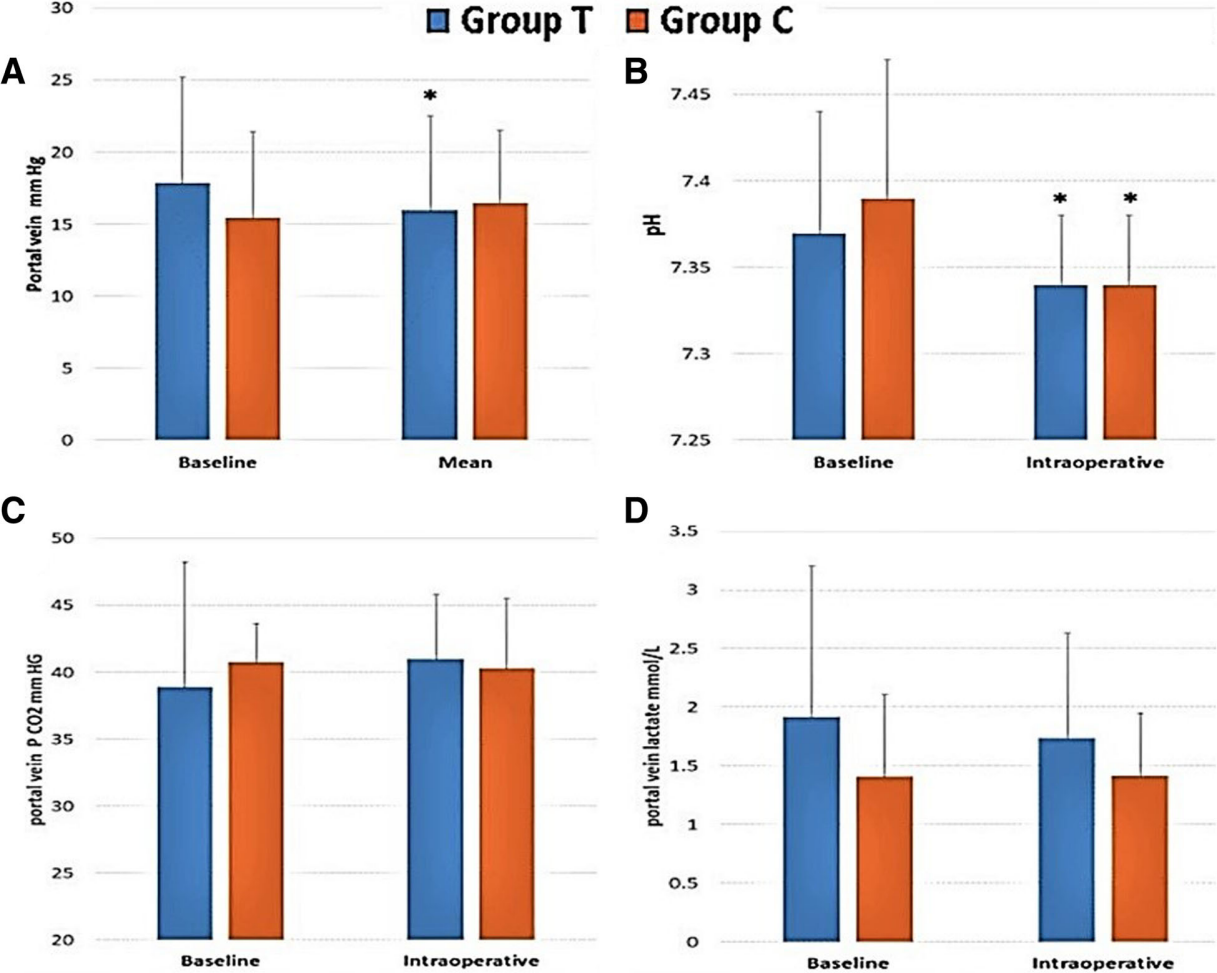
Intraoperative changes in the portal vein. Caption; (**a**)portal venous pressure, (**b**) Portal venous pH, (**c**) Portal venous partial pressure of CO2, (**d**) portal venous blood lactate level. Data are presented as mean ± SD. (*) significant change from the baseline value. *P*-value < 0.05 was considered statistically significant

Baseline values of portal pressure in both groups showed an insignificant difference (*P* = 0.21). The initiation of terlipressin infusion was accompanied by a significant reduction of portal pressure from 17.88 ± 7.32 mmHg at baseline to 15.96 ± 6.55 mmHg (*p* < .001) in terlipressin, whereas in the control group, there was an insignificant change of portal pressure from 15.48 ± 5.92 mmHg at baseline to 16.48 ± 5.04 mmHg (*p* = 0.52). No significant difference was observed between both groups concerning the mean intraoperative values of portal pressure (Fig. 2-a).

As regards to the portal vein pH, PCO2 and lactate, the pH only showed significant decrease in each group selectively during the intraoperative period when compared to the corresponding baseline value there were insignificant differences between the two groups, otherwise, insignificant changes and differences were noticed as regard PCO2, and lactate level in the portal vein blood (Fig. 2-b, c, d).

Hemodynamic changes are shown in (Table 2). There was a significant increase of intraoperative SVR as compared to its baseline value in the terlipressin group only (*p* = .040), and there was a significantly higher intraoperative SVR in terlipressin group in comparison to the control group (Table 2). Three patients in the control group versus only one patient in the terlipressin treated group required norepinephrine as a vasopressor with no one needed dobutamine, but this was statistically insignificant (Table 2).

**Table 2:** Hemodynamic variables

Variables		Group T N = 25	Group C N = 25	*P* value
HR (beat/m.)	Baseline	77.3 ± 7.9	80.1 ± 7.7	0.22
	Intraoperative	79.6 ± 14.4	84.5 ± 14.5	0.24
	Postoperative	85.2 ± 11.3*	90.8 ± 12*	0.09
MAP (mm HG)	Baseline	85.8 ± 8.7	89.4 ± 12.8	0.25
	Intraoperative	88.7 ± 7.2	83.9 ± 6.98	0.02^¥^
	Postoperative	80.2 ± 12.3	86.1 ± 9.9	0.07
CVP (mm Hg)	Baseline	14.0 (8–20)	14.0 (8–22)	0.34
	Intraoperative	12.4 (8.3–19.2)	12.6 (7.1–21.4)	0.84
	Postoperative	9.0 (2–12)”	8.0 (5–21)”	0.60
CI (L/min/m^2^)	Baseline	3.6 ± 0.9	3.4 ± 1.1	0.43
	Intraoperative	3.1 ± 0.3*	3.3 ± 0.6	0.15
SVV %	Baseline	7.4 ± 3.90	9.2 ± 2.98	0.08
	Intraoperative	10.6 ± 2.17*	11.0 ± 2.14*	0.50
SVR (dyne.s/cm^5^)	Baseline	1030.2 ± 318.1	1021.8 ± 285.2	0.92
	Intraoperative	1144.9 ± 221.0*	1007.2 ± 232.4	0.04^¥^
Need for inotropic support, no. of patients		1(4%)	3(12%)	0.30
Inotropic score		5 (5–5)	10(5–10)	0.32

Data are presented as mean ± SD or median (range). HR heart rate; MAP mean arterial pressure; CI cardiac index; SVV stroke volume variation; SVR systemic vascular resistance. *P*-value <0.05 was considered statistically significant. (*) significant difference in comparison to the baseline value within the same group. (^¥^) significant difference between the two groups. The inotropic score is calculated as follows: One point was assigned for each mcg/kg/min of dobutamine, and 1 point was assigned for each 10 ng/kg/min of norepinephrine)

The mean arterial blood pressure was significantly higher in group T than in group C during the intraoperative period, with non-significant changes compared to the baseline values in both groups. No significant difference was observed between groups concerning HR, CO, CI, and SVV. There was a significant reduction in CI when compared to its baseline value in terlipressin group only (*P* = 0.002) (Table 2).

Urine output was significantly higher in the T group than C group during the intraoperative period and the first three postoperative days; however, insignificant differences were noted between both groups as regard to blood urea, creatinine, sodium, and potassium. There was no significant difference in the total number of patients who developed renal complications (Acute Kidney Injury) (Table 3).

**Table 3:** Renal and electrolytes changes

variables	Timing	Group T N = 25	Group C *N* = 25	*P* value
Urine output (ml/kg/hr.)	Intraoperative	1.88 (0.6–8.7)	1.12 (0.5–3.8)	0.017^¥^
	1st postoperative day	1.00 (0.60–5.70)	0.93 (0.51–2.80)	0.029^¥^
	2nd postoperative day	1.25 (0.60–4.90)	1.10 (0.48–2.00)	0.048^¥^
	3rd postoperative day	2.00 (1.00–4.60)	1.20 (0.60–3.60)	0.005^¥^
Urea (mmol/L)	Preoperative	4.4 (3.0–7.5)	5.1 (1.7–16.8)	0.75
	1st postoperative day	3.9 (2.3–8.8)	5.0 (1.9–11.0)	0.23
	2nd postoperative day	5.9 (3.2–15.0) *	5.3 (2.1–14.0)	0.39
	3rd postoperative day	5.6 (3.3–17.0) *	5.3 (3.3–18.0)	0.59
Creatinine (μmol/L)	Preoperative	62.8 **±** 11.2	73.5 **±** 28.6	0.09
	1st postoperative day	58.3 **±** 20.7	68.7 **±** 20.5	0.08
	2nd postoperative day	65.4 **±** 21.4	77.2 **±** 40.0	0.20
	3rd postoperative day	60.6 **±** 15.8	66.0 **±** 33.5	0.42
Na^+^ (mmol/L)	Preoperative	137.7 **±** 3.9	139.5 **±** 3.8	0.10
	1st postoperative day	137.8 **±** 4.3	139.8 **±** 3.3	0.08
	2nd postoperative day	137.4 **±** 2.8	137.9 **±** 6.6	0.75
	3rd postoperative day	137.0 **±** 2.9	138.2 **±** 4.1	0.27
K^+^ (mmol/L)	Preoperative	3.7 **± 0**.4	3.8 **±** 0.5	0.16
	1st postoperative day	3.7 **±** 0.6	3.9 **±** 0.6	0.22
	2nd postoperative day	3.5 **± 0**.4	3.6 **±** 0.7	0.74
	3rd postoperative day	3.4 **±** 0.6*	3.5 **±** 0.6*	0.51
AKI incidence		1 (4%)	2(8%)	0.51

Data are presented as mean ± SD or median (range). AKI acute kidney injury. P-value < 0.05 was considered statistically significant. (*) significant difference in comparison to the baseline value within the same group. (¥) significant difference between the two groups

Estimated intraoperative blood loss was significantly higher in the control group in comparison to terlipressin group (*p* = 0.004); consequently, colloids and the units of transfused packed RBCs were significantly higher in the control group respectively (*P* = 0.03, *p* = 0.003) (Table 4).

**Table 4:** Fluids and blood products transfusion

Variable	Group T N = 25	Group C N = 25	*P* value
Crystalloids (ml)	4000 (2000–6500)	4500 (1500–6500)	0.88
Colloids (ml)	182 ± 142	284 ± 188	0.03¥
Packed RBCs (units)	0.0 (0–2)	1.0 (0–4)	0.003¥
FFP (units)	0.5 (0–4)	0.0 (0–3)	0.76
Estimated blood loss (ml)	842 ± 145.5	1065.7 ± 202	0.004¥
Hemoglobin (gm/dl)			
Preoperative	12.32 ± 1.23	13.07 ± 1.67	0.07
1st postoperative day	12.28 ± 1.54	11.79 ± 1.85*	0.3

Data are presented as mean ± SD or median (range). FFP fresh frozen plasma. P-value < 0.05 was considered statistically significant

## Discussion

In the present study, intraoperative terlipressin infusion was used during hepatobiliary surgery in a trial to obtain a better portal and systemic hemodynamics. Terlipressin administration was accompanied by a significant (within the same group) decrease of portal pressure. Terlipressin works through (V1) receptors in the vascular smooth muscle in the splanchnic blood vessels, which induces vasoconstriction and reduction in arterial blood flow to the splanchnic area with subsequent reduction in portal blood flow and thus a reduction in portal blood pressure [11]. Elevated portal pressure can complicate liver resection by increasing the risk of hemorrhage, liver failure, varices rupture, and coagulation disorders caused by thrombocytopenia [13].

Previous studies have demonstrated that intravenous injection of terlipressin improves renal function in hepatorenal syndrome patients, and at the same time can affect splanchnic and systemic circulations in cirrhotic patients [14, 15]. However, no previous studies have investigated the effects of intraoperative administration of terlipressin on systemic and hepatic hemodynamics or renal function in patients undergoing major hepatobiliary surgery.

Fahrner and his colleagues reported better liver regeneration due to reduced portal venous pressure through terlipressin administration during the postoperative period [16]. A randomized controlled trial was done by Reddy and co-workers documented that perioperative terlipressin administration during living donor liver transplantation reduces intraoperative portal venous pressure and decreases the incidence of postoperative acute kidney injury [17].

Wagener’s study had mentioned that vasopressin administration could decrease portal vein pressure as well as the flow in the native liver without reducing cardiac output or intestinal perfusion in liver transplant patients [18]. Mukhtar and others demonstrated that intraoperative terlipressin infusion improved mean arterial pressure and systemic vascular resistance with an insignificant reduction in cardiac output, heart rate, or oxygen consumption [12]. In a similar study, Fayed and his colleges reported a significant improvement in the MAP, but with an associated decrease in CO and HR [19]. Kalambokis and others studied the effects of terlipressin on hemodynamics in cirrhotic liver patients and observed increases in SVR [20]. In the current study, terlipressin infusion was associated with a significant increase in intraoperative SVR and MAP in comparison to the control group, and there was a significant reduction in CI when compared to the baseline in terlipressin group; however, these changes remained within the accepted clinical ranges.

Our results as regards the improvement in SVR and the higher mean blood pressure in the terlipressin group are in agree with Hong and his colleagues’ study where they utilized terlipressin infusion in a dose of 1.0–4.0 μg/kg per hour in living donor liver transplant. They noted that terlipressin infusion was associated with increased SVR, and this offered better postoperative renal function, and shorter duration in ICU in comparison to patients who did not receive terlipressin [21].

Many side effects to the use of terlipressin has been detected in different studies. Intestinal hypoperfusion is one of these side effects that might be noticed in patients with esophageal variceal hemorrhage, and portal hypertension who are treated with terlipressin [22, 23]. Same while, Wagener and his colleagues documented that vasopressin infusion during liver transplantation was associated with a reduction in portal pressure with no signs of splanchnic hypoperfusion [18]. In our study, terlipressin managed to decrease portal venous pressure significantly without any signs of intraoperative splanchnic hypoperfusion in the form of increased portal PCO2 or lactate and portal venous pH remained within normal.

In the past, terlipressin was used to be administered by intermittent intravenous bolus injections. Recently, continuous infusion of (1.3–2.6 μg/kg/hr) of terlipressin early in distributive shock was used. This protocol significantly increased SVR with no undesirable side-effects, such as an excessive elevation in peripheral vascular resistance [24, 25]. The dose used in our study was within the range of doses previously used for low-dose infusion.

In our study, one of the benefits of intraoperative terlipressin infusion in such major prolonged surgeries was the significant reduction of estimated blood loss in the terlipressin group which was reflected in the form of a significant reduction in the transfused packed RBCs units. This is in agreement with our previous study on liver resection surgeries [26] Fayed and colleagues who showed that intraoperative blood loss during living donor liver transplant was significantly lower in the terlipressin group than in the control (placebo) group (2212.5 mL vs. 2787.5 mL, respectively; P <0.05) [19]. Raedler’s study showed that vasopressin reduced bleeding and improved outcome after blunt liver trauma and uncontrolled hemorrhagic shock in a pig model [27].

It has been documented that terlipressin administration can decrease plasma concentrations of renin, aldosterone, and norepinephrine. This reduction in such vasoconstrictors increases renal blood flow and improves kidney function [14, 18, 21, 23, 28–30]. In our study, terlipressin group patients have a significant increase in UOP compared with controls, but no significant change in serum Creatinine was observed. This may be assumed to the enhancement of renal perfusion by increasing both mean arterial pressure and effective arterial blood volume through arteriolar vasoconstriction in the splanchnic area and by redistributing blood to the systemic circulation [14]. Stimulation of V1a receptors may mediate another mechanism of increased diuresis [31]. The incidence of postoperative complications including renal dysfunction in patients given intraoperative terlipressin infusion was not significantly different from the control.

It was mentioned in a retrospective study done by Yim and co-workers that the risk factors for the development of hyponatremia with terlipressin administration are young age patients, low body mass index, longer duration of terlipressin administration, and high base values of serum sodium which was not the case in our study [32].

### Limitations

one limitation is that we have included patients with and without portal hypertension. Also, the use of colloid solution (Voluven) was not the ideal especially when we study renal function, but in our country, we don’t have other colloid options. One more limitation is that we did not consider the influence of terlipressin on renal tubular function through the sensitive markers, e.g., cystatin c, and urinary neutrophil gelatinase-associated lipocalin and our results about renal parameters were not correlated with postoperative fluid intake and balance. Another limitation of this study is that we didn’t document other side effects of terlipressin as limb ischemia and postoperative mortality.

## Conclusion

intraoperative infusion of terlipressin is shown to be an effective technique for reduction of portal venous pressure, and blood loss with better maintenance of systemic mean blood pressure and no significant sign of intestinal ischemia in patients undergoing major hepatobiliary surgery.

## Data Availability

The datasets generated and/or analyzed during the current study are not publicly available due to the regulation of our institution, but are available from the corresponding author after getting permission from the institution for sharing the dataset on reasonable request.

## Abbreviations

ASA: American Society of Anesthesiologists
CI: Cardiac Index
CONSORT: Consolidating Standards of Reporting Trials
COP: Cardiac Output
CVP: Central Venous Pressure
Hb: Hemoglobin level
HR: Heart Rate
ICU: Intensive Care Unit
MAP: Mean arterial blood pressure
SVR: Systemic Vascular Resistance
SVV: Stroke Volume Variation
UOP: Urine output
